# Substrate Oxidation by Indoleamine 2,3-Dioxygenase: EVIDENCE FOR A COMMON REACTION MECHANISM*

**DOI:** 10.1074/jbc.M115.695684

**Published:** 2015-10-28

**Authors:** Elizabeth S. Booth, Jaswir Basran, Michael Lee, Sandeep Handa, Emma L. Raven

**Affiliations:** From the ‡ Department of Chemistry, University of Leicester, University Road, Leicester LE1 7RH, Great Britain, United Kingdom and; § Department of Molecular and Cellular Biology and Henry Wellcome Laboratories for Structural Biology, Henry Wellcome Building, University of Leicester, Lancaster Road, Leicester LE1 9HN, Great Britain, United Kingdom

**Keywords:** dioxygenase, enzyme mechanism, heme, substrate specificity, tryptophan, indoleamine 2,3-dioxygenase

## Abstract

The kynurenine pathway is the major route of l-tryptophan (l-Trp) catabolism in biology, leading ultimately to the formation of NAD^+^. The initial and rate-limiting step of the kynurenine pathway involves oxidation of l-Trp to *N*-formylkynurenine. This is an O_2_-dependent process and catalyzed by indoleamine 2,3-dioxygenase and tryptophan 2,3-dioxygenase. More than 60 years after these dioxygenase enzymes were first isolated (Kotake, Y., and Masayama, I. (1936) *Z. Physiol. Chem.* 243, 237–244), the mechanism of the reaction is not established. We examined the mechanism of substrate oxidation for a series of substituted tryptophan analogues by indoleamine 2,3-dioxygenase. We observed formation of a transient intermediate, assigned as a Compound II (ferryl) species, during oxidation of l-Trp, 1-methyl-l-Trp, and a number of other substrate analogues. The data are consistent with a common reaction mechanism for indoleamine 2,3-dioxygenase-catalyzed oxidation of tryptophan and other tryptophan analogues.

## Introduction

**SCHEME 1.**
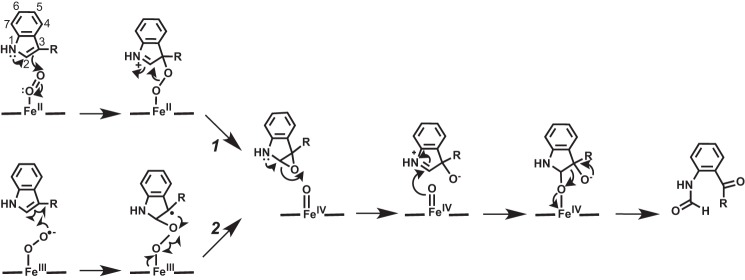
**Possible mechanisms for tryptophan oxidation in the heme tryptophan dioxygenases.** Route *1* shows an electrophilic mechanism. Route *2* shows a radical addition mechanism.

**SCHEME 2.**
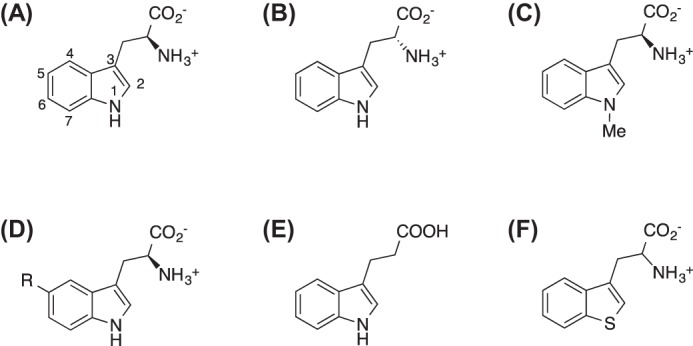
**Structures of the tryptophan analogues used in this study.** *A*, l-Trp; *B*, d-Trp; *C*, 1-Me-l-Trp; *D*, general structure for the series of 5-substituted tryptophan analogues (R = Me (5-Me-dl-Trp), R = OH (5-OH-l-Trp), R = OMe (5-MeO-Trp), R = F (5-F-l-Trp)); *E*, IPA; *F*, S-Trp.

The heme-containing tryptophan dioxygenase enzymes, indoleamine 2,3-dioxygenase (IDO)^2^ and tryptophan 2,3-dioxygenase (TDO), catalyze the O_2_-dependent oxidation of tryptophan to *N*-formylkynurenine (NFK). This reaction is the first step in the kynurenine pathway, which leads to the formation of NAD^+^ (1). The mechanism of tryptophan oxidation has taken many years to establish and is still not clarified (2–4). The first step had been proposed almost 50 years ago (5) as a base-catalyzed proton abstraction and was based on the assumption that a suitable base (presumed to be a histidine) was present in the active site. This mechanism also assumed that the only reactive substrates were those containing a hydrogen atom on the indole nitrogen of l-tryptophan (l-Trp; Scheme 1). But we have demonstrated (6) that the 1-methyl-l-Trp analogue (1-Me-Trp; Scheme 2) is also a substrate for human IDO (hIDO) (others report similar findings (7)). Because base-catalyzed abstraction of a Me group is chemically impossible, we suggested (6) that the reaction mechanism needed reassessment.

Although support for the early mechanistic proposals (5) has recently waned, what happens instead is not known across the family of IDO and tryptophan 2,3-dioxygenase enzymes. Later steps of the mechanism are only partly clarified. Formation of a transient Compound II (ferryl) intermediate is implicated from resonance Raman (8, 9) data, and computational work supports this (10, 11). However, the evidence for ferryl heme formation during turnover has so far been limited to IDO (with no evidence as yet for tryptophan 2,3-dioxygenase) and for only one substrate (l-Trp). There is no mechanistic information on the reactivity of IDO with any substrates other than l-Trp and therefore no indication of whether other substrates react by the same mechanism.

These questions lie at the heart of the debate on heme dioxygenase reactivity. It is important in the context of drug discovery programs because IDO has a wide substrate specificity and has attracted considerable interest as a therapeutic target in neurological disease and cancer (12–14), and there is commercial interest in the search for IDO inhibitors (with 1-Me-Trp already in clinical trials). The aim of this work was therefore to examine the mechanism of IDO-catalyzed oxidation across a range of Trp substrates and to establish whether all react using a common mechanism.

## Experimental Procedures

### 

#### 

##### Materials

All chemicals used in this work were purchased from Sigma-Aldrich and were of the highest purity (>99% purity), except for 1-methyl-l-tryptophan (1-Me-l-Trp; 95% purity) which is contaminated with l-Trp and requires further purification by HPLC as noted previously (6). l-Trp, d-tryptophan (d-Trp), 1-Me-l-Trp, indole-3-propionic acid (IPA), 5-hydroxy-l-tryptophan (5-OH-l-Trp), 5-fluoro-l-tryptophan (5-F-l-Trp), and 5-methoxy-dl-tryptophan (5-MeO-Trp) were purchased from Sigma-Aldrich; 5-methyl-dl-tryptophan (5-Me-dl-Trp) was purchased from Acros Organics; and β-[3-benzo(*b*)thienyl]-l-alanine (S-Trp) was purchased from Apollo Scientific. Scheme 2 gives the structures of all substrates used in this work.

##### Preparation of IDO

hIDO was purified as described previously (15, 16), and the protein concentration was determined from the reported absorption coefficient (hIDO ϵ_404_ = 172 mm^−1^ cm^−1^).

##### Kinetics

Pre-steady state stopped-flow experiments were carried out using an Applied Photophysics SX.18MV stopped-flow spectrometer housed in an anaerobic glove box (Belle Technology Ltd.; [O_2_] < 5 ppm) and fitted with a Neslab RTE-200 circulating water bath (25.0 ± 0.1 °C). In stopped-flow experiments, stated concentrations of protein and reagents relate to final concentrations in the flow (after mixing). Detection of Compound II under turnover conditions (*i.e.* in the presence of O_2_ and substrate) was observed in sequential mixing mode, monitoring absorbance changes at 593 nm that report on the formation and decay of the ferryl species without complication from any other absorbing species. Spectral deconvolution was performed by global analysis and numerical integration methods using Pro-Kineticist software (Applied Photophysics Ltd.). The experiment was initiated by mixing ferrous enzyme (10 μm; generated by stoichiometric titration of ferric enzyme with sodium dithionite) with oxygen-saturated buffer (50 mm Tris-HCl, pH 8.0, [O_2_] = 1.2 mm) and then aging the solution for 50 ms to ensure complete formation of Fe(II)-O_2_ before a second mix with l-Trp or tryptophan analogues ([Trp] ≥ 10 × *K_m_* in cases where *K_m_* is known; Table 1). Formation and decay of ferryl heme or NFK was followed at 593 or 321 nm, respectively (except for the case of 5-methoxy-dl-tryptophan where the wavelength maximum for product formation was at 354 nm). In cases where substrate was present in excess, decay of Compound II led to formation of ferrous heme at the end of the experiment except where [substrate] was low in which case decay to ferric heme was observed instead (consistent with the reported increase in reduction potential in the presence of substrate (17, 18)). In other, non-turnover reactions, ferrous hIDO (2.5 μm) was mixed with H_2_O_2_ (5 eq) in either single mixing mode or with H_2_O_2_/substrate in sequential mixing mode. Steady-state assays (50 mm Tris-HCl buffer, pH 8.0, 25.0 °C) measuring formation of NFK at 321 nm were performed in solutions containing 20 mm l-ascorbate, 10 μm methylene blue, 100 μg of catalase, and a fixed concentration of enzyme (≈100 nm or less) according to published protocols (16).

##### Mass Spectrometry

For mass spectrometry experiments, formation of product or the intermediate 2,3-epoxide species was carried out in a glove box ([O_2_] < 5 ppm) by incubation of ferrous enzyme (0.5–1 μm; generated by stoichiometric titration of ferric enzyme with ≤2 eq of dithionite) with either l-Trp or a tryptophan analogue ([Trp] ≥ 10 × *K_m_* in cases where *K_m_* is known; Table 1) prior to addition of aerobic solutions ([O_2_] = 258 μm) of buffer (50 mm Tris-HCl, pH 8.0) (19). Samples were allowed to react for varying amounts of time (15–60 min) before being centrifuged (13,000 rpm, 3 min), and the supernatant was frozen directly on dry ice. Samples were stored at −80 °C until required for LC-MS analysis.

## Results

### Detection of a Compound II Intermediate during Steady-state Oxidation of l-Trp by IDO

Identification of the reaction intermediates in IDO has been difficult (see “Discussion”). We designed an anaerobic stopped-flow experiment to cleanly differentiate between ferrous-oxy species and the transient intermediates formed later in the catalytic cycle by forming the ferrous-oxy species in high (>95%) yield prior to addition of substrate to initiate turnover.

### Reaction with l-Trp

**FIGURE 1.**
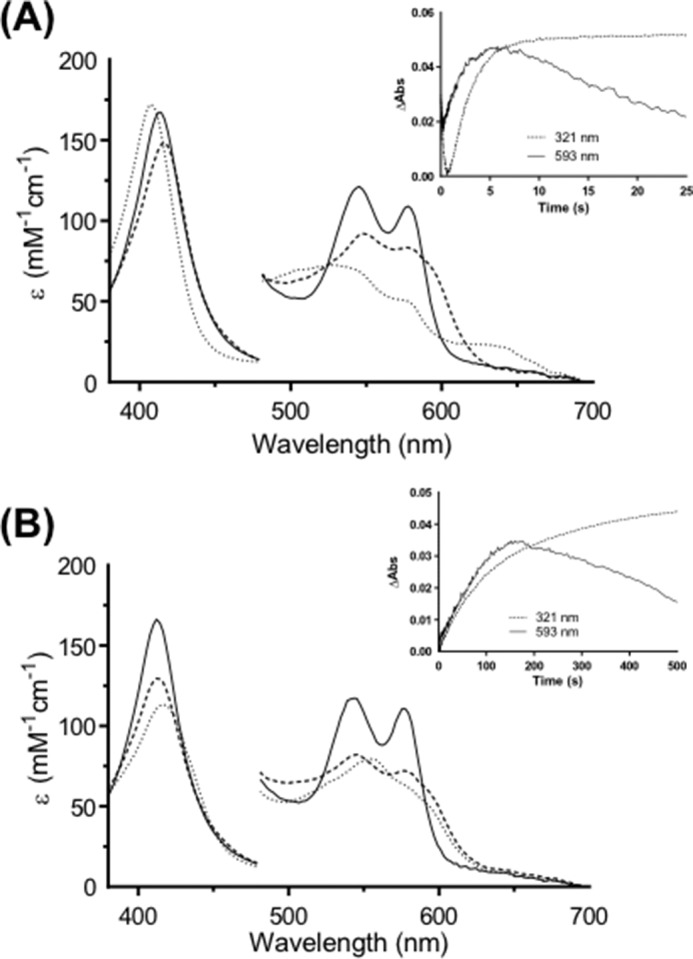
**Formation of a ferryl intermediate during oxidation of l-Trp and 1-Me-l-Trp by ferrous hIDO.** Stopped-flow diode array spectra are shown. The *solid line* in each case is the first spectrum recorded after mixing and represents the ternary [Fe(II)-O_2_, substrate] complex, the *dashed line* is Compound II, and the *dotted line* represents the final spectrum. Ferrous hIDO (2.5 μm) was premixed with O_2_ (300 μm) for 50 ms followed by mixing with l-Trp (50 μm) and monitored over 100 s (*A*) or 1-Me-l-Trp (1.5 mm) and monitored over 500 s (*B*). Absorbance (*Abs*) values in the visible region have been multiplied by a factor of 5. *Insets* show absorbance changes that report on NFK (at 321 nm) and Compound II (at 593 nm; absorbance changes multiplied by a factor of 20) formation and decay.

**FIGURE 2.**
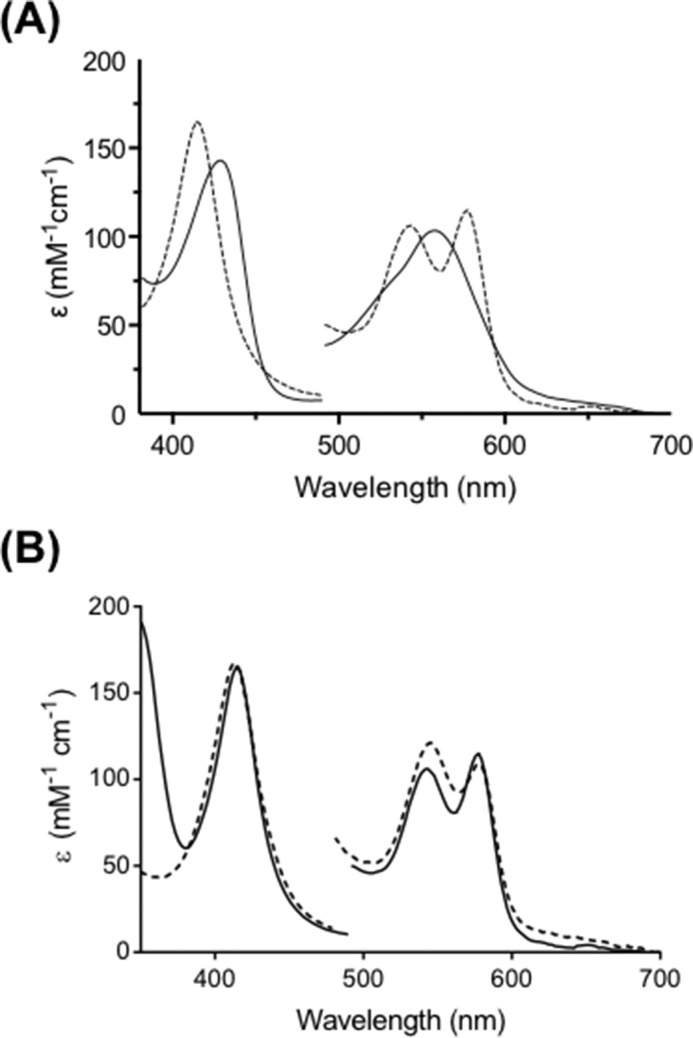
**Formation of ferrous-oxy hIDO and comparison with the ternary complex.** *A*, Stopped-flow spectra showing the formation of ferrous-oxy hIDO. Ferrous hIDO (2.5 μm) was mixed with O_2_-saturated buffer. The *solid line* is ferrous hIDO, and the *dashed line* is ferrous-oxy hIDO. *B*, comparison of the spectra of ferrous-oxy IDO (Fe(II)-O_2_) species (*solid line*; from *A*) and that of the ternary [Fe(II)-O_2_, Trp] complex in IDO (*dashed line*; from Fig. 1*A*). Absorbance values in the visible region have been multiplied by a factor of 5.

Under anaerobic stopped-flow conditions, ferrous hIDO was incubated for 50 ms with O_2_-saturated buffer, to allow for complete formation of the Fe(II)-O_2_ complex, prior to a second mix with l-Trp. The first spectrum (at 4 ms after mixing with l-Trp) is consistent with formation of a ternary [Fe(II)-O_2_, l-Trp] complex (λ_max_ = 413, 543, and 577 nm; Fig. 1*A*), although the α/β ratio (<1) and the Soret band (at 413 nm) are very slightly different from a “pure” sample of ferrous-oxy prepared in the same way but without the second mix (α/β ratio = 1.06, λ_max_ = 416, 543, and 577 nm; Fig. 2*A*). An overlay of the spectra of the oxy and ternary complexes is shown in Fig. 2*B*; these alignments of the oxy and ternary spectra allow identification of the intermediate ferryl species below.

After a lag phase of 0.5 s, NFK formation was observed at 321 nm (Fig. 1*A*, *inset*). During NFK formation, an intermediate accumulated (λ_max_ = 416, 547, 577, and 593^sh^ nm; Table 1) as evidenced by the appearance of a shoulder in the visible region (absorbance increases at 593 nm; Fig. 1, *A* and *inset*). This intermediate was assigned as arising from a Compound II species. Formation of a ferryl (Compound II) species has been suggested previously from resonance Raman work (8), and there is agreement on this point (9) (a similar species to that detected in Fig. 1 has been observed previously under similar conditions (9), but its identity was not confirmed). In our experiments, the Compound II species existed during NFK production over 5 s and then decayed when l-Trp was depleted and NFK production ceased. Compound II formation (measured at 593 nm; Fig. 1*A*, *inset*) correlates exactly with NFK production and decay (measured at 321 nm), which is clear evidence that Compound II is an intermediate in the mechanism and that its decay is rate-limiting.

**TABLE 1:** **Summary of kinetic and turnover data for hIDO with various substrates from steady-state (k_cat_ and K_m_) and pre-steady-state (Compound II maxima) experiments** NFK formation was observed (by LC-MS and by increases in absorbance at 321 nm) for all substrates except for S-Trp. ND, not detected.

Substrate/analogue	hIDO				
	*k*_cat_	*K_m_*	Compound II λ_max_	LC-MS	
				Epoxide	Product
	*s*^−*1*^	μ*m*	*nm*		
l-Trp	1.4 ± 0.1*^a^*	7.0 ± 0.8*^a^*	416, 547, 577, 593^sh^	+	+
1-Me-l-Trp	0.027 ± 0.001*^b^*	150 ± 11*^b^*	413, 543, 577, 593^sh^	+*^c^*	+
5-F-Trp	0.76 ± 0.01*^a^*	6.0 ± 0.8*^a^*	414, 546, 576, 593^sh^	+	+
5-Me-Trp	3.8 ± 0.2*^a^*	98 ± 14*^a^*	413, 547, 576, 593^sh^	+	+
d-Trp	3.9 ± 0.1*^a^*	1600 ± 100*^a^*	413, 546, 578, 593^sh^	+	+
5-OH-Trp	0.025 ± 0.001*^a^*	17 ± 1*^a^*	ND	−	+
5-MeO-Trp	0.78 ± 0.06	40 ± 16	413, 545, 577, 593^sh^	+	+
IPA	—*^d^*	—*^d^*	ND	+	+
S-Trp	—*^e^*	—*^e^*	413, 546, 577, 593^sh^	+	−

*^a^* Kinetic constants taken from Ref. 28.

*^b^* Kinetic constants taken from Ref. 6.

*^c^* Epoxide formation reported in Ref. 19 and 28.

*^d^* There are no reports of steady-state rate constants for IPA in the literature likely because the increased enzyme and substrate concentration needed to observe turnover lead to higher background absorbances.

*^e^* Kinetic parameters for S-Trp have not been reported previously and could not be determined from steady state assays in this work because there are no changes in absorbance at 321 nm with this substrate.

**FIGURE 3.**
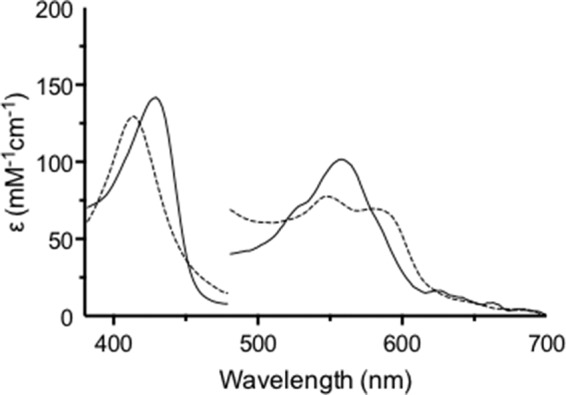
**Formation of Compound II from reaction with peroxide.** Stopped-flow spectra show the formation of Compound II monitored over 5 s. Ferrous hIDO (2.5 μm; *solid line*) was premixed with H_2_O_2_ (5 eq) for 50 ms followed by mixing with l-Trp (50 μm). The *dashed line* is Compound II. Absorbance values in the visible region have been multiplied by a factor of 5.

To verify the identity of the intermediate species identified in the above reactions, we carried out experiments in which Compound II was prepared directly, under similar anaerobic conditions, but by reaction of ferrous heme with peroxide (a method previously used for preparation of Compound II in cytochrome *c* peroxidase (20)). Reaction of ferrous hIDO with H_2_O_2_ (5 eq) in the presence of l-Trp (Fig. 3) shows conversion of the ferrous species (λ_max_ = 425, 527^sh^, and 558 nm) to a second species that has wavelength maxima (λ_max_ = 413, 547, 579, and 593^sh^ nm) that are essentially identical to the species observed under turnover conditions above (Fig. 1).

### Detection of a Compound II Intermediate during Turnover of hIDO with Other Substrates

#### 

##### S-Trp

Along with 1-Me-l-Trp (examined below) and O-Trp, S-Trp was originally reported (21) as an inhibitor of IDO. This was rationalized by assuming a base-catalyzed abstraction mechanism (which is not possible with S-Trp, hence the inhibition). But 1-Me-l-Trp is now reclassified as a slow substrate (6), and this is so far the main experimental evidence used to rule out the base-catalyzed abstraction mechanism (assuming that all substrates react by a common mechanism). Because of these ambiguities in the assignments of reactivities for IDO substrates, the activity of S-Trp was reassessed.

**FIGURE 4.**
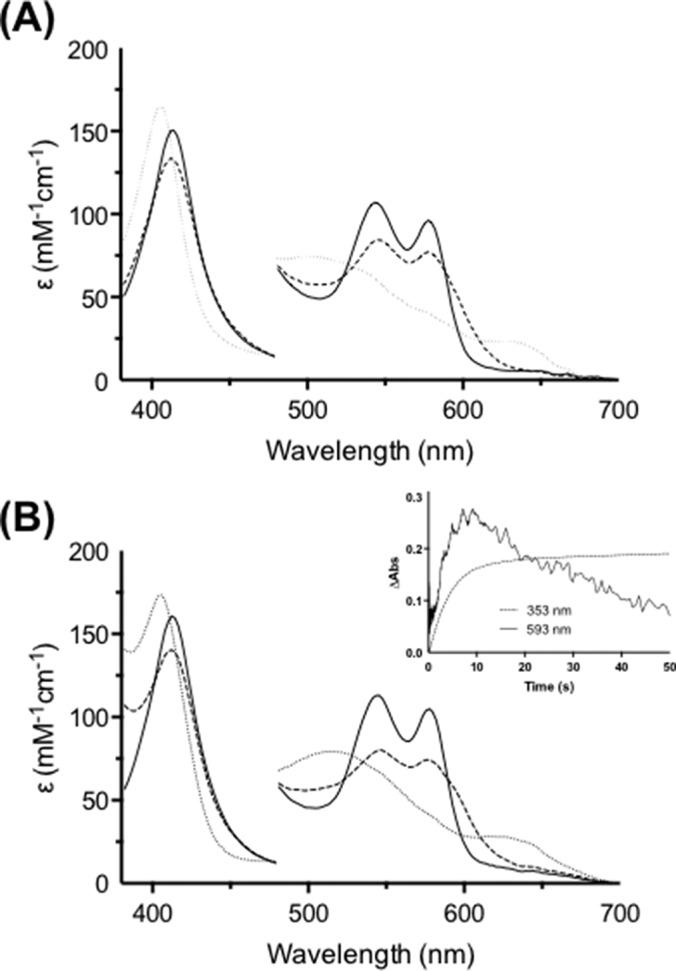
**Formation of a ferryl intermediate during oxidation of S-Trp and 5-MeO-Trp by ferrous hIDO.** Stopped-flow diode-array spectra are shown. Ferrous hIDO (2.5 μm) was premixed with O_2_ (300 μm) for 50 ms followed by mixing with S-Trp (200 μm; monitored over 200 s) (*A*) or 5-MeO-Trp (250 μm; monitored over 100 s) (*B*). In *A* and *B*, the *solid line* is the proposed ternary [enzyme-O_2_-substrate] complex, the *dashed line* is assigned as Compound II, and the *dotted line* is the final spectrum. Absorbance (*Abs*) values in the visible region have been multiplied by a factor of 5. The *inset* in *B* shows absorbance changes that report on NFK (354 nm) and Compound II (593 nm; absorbance changes at 593 nm have been multiplied by a factor of 20). Absorbance (*Abs*) values in the visible region have been multiplied by a factor of 5.

For S-Trp, formation of a ternary [Fe(II)-O_2_, S-Trp] species was observed (Fig. 4*A*, *solid line*) prior to formation of an intermediate species which, by analogy with the data for l-Trp above, was assigned as Compound II (λ_max_ = 413, 546, 577, and 593^sh^ nm; Fig. 4*A*). These kinetic data provide convincing evidence against base-catalyzed abstraction because formation of Compound II is not possible if the indole NH of the substrate is replaced with a sulfur atom; it is instead consistent with either of the two mechanisms shown in Scheme 1.

We have previously used mass spectrometry to identify products formed during IDO-catalyzed oxidations (19). In separate experiments on S-Trp using mass spectrometry, we observed evidence for formation of the corresponding 2,3-epoxide (*m*/*z* = 238), but product formation (*m*/*z* = 254) was not detected by mass spectrometry or in steady-state assays (at 321 nm) probably because the alignment of the lone pairs on sulfur do not favor the ring-opening step leading to product formation (Scheme 1). Identification of an epoxide is consistent with the formation of a ferryl intermediate during the mechanism (Scheme 1).

##### 1-Me-l-Trp

In an identical stopped-flow experiment as that carried out for l-Trp above, we also observed formation of a ternary [Fe(II)-O_2_, 1-Me-l-Trp] complex (λ_max_ = 413, 545, and 577 nm; Fig. 1*B*). After a lag phase of 4 s, NFK formation was observed (Fig. 1*B*, *inset*), and an intermediate accumulated (λ_max_ = 413, 543, 577, and 593^sh^ nm) as evidenced by the appearance of the shoulder at 593 nm and increases in absorbance at this wavelength (Fig. 1, *B* and *inset*) that persisted over 150 s. We assigned this as a Compound II. As in the case of l-Trp, formation of the Compound II (monitored at 593 nm) is correlated to NFK production (at 321 nm), which confirms a role for Compound II as an intermediate in the oxidation of 1-Me-Trp.

We conclude that oxidation of l-Trp and 1-Me-l-Trp by ferrous hIDO proceeds via the same intermediate. This is the first evidence that other substrates react via the same oxidative mechanism as that used for l-Trp and that a common reaction intermediate (Compound II) is observed.

##### 5-F-Trp, 5-Me-Trp, and d-Trp

We extended the scope of our study to examine the reactivity of hIDO with other substrates. To date, there is no mechanistic information available for these substrates. Data for all substrates, including steady-state activities (determined separately), are summarized in Table 1.

Parallel experiments were carried out with 5-F-Trp, 5-Me-Trp, and d-Trp (Scheme 2), and these experiments identified the same intermediate as observed for l-Trp and 1-Me-l-Trp (Table 1). This is the first observation of a Compound II intermediate for these numerous substrates, and the data are consistent with all substrates reacting by the same mechanism.

##### Probing the Reactivity of Other Substrates by IDO

Further information was extracted from reaction of hIDO with three other substrates, 5-OH-l-Trp, 5-MeO-Trp, and IPA (Scheme 2).

##### 5-OH-l-Trp and 5-MeO-Trp

On reaction of ferrous hIDO with O_2_ and then 5-OH-l-Trp, formation of a ternary complex ([Fe(II)-O_2_, 5-OH-l-Trp]) was initially observed (λ_max_ = 413, 543, and 577 nm; data not shown). No evidence for formation of a Compound II intermediate was observed, and there were no significant increases in absorbance at 321 nm that could be attributed to NFK formation. In steady-state assays, however, product formation at 321 nm was observed, although 5-OH-Trp is a very slow substrate (*k*_cat_ = 0.025 s^−1^; Table 1).^3^ LC-MS analysis of the steady-state reaction products identified a product (*m*/*z* = 253); however, there was no evidence for the formation of a 2,3-epoxide as has been observed (19) for l-Trp. If the mechanism of oxidation of 5-OH-l-Trp proceeds by radical addition (Scheme 1, Route 2), then in the case of 5-OH-l-Trp radical formation at C^3^ could lead to hydrogen atom abstraction from the 5-OH group and a failure of Compound II to accumulate as in the case of the other substrates. We tested this hypothesis using 5-MeO-Trp in which hydrogen atom abstraction from the 5-OH group is not possible. In contrast to 5-OH-l-Trp, the 5-MeO-Trp is a good substrate (*k*_cat_ = 0.8 s^−1^).^4^ Furthermore, oxidation of 5-MeO-Trp by ferrous hIDO clearly showed formation of a Compound II intermediate (Fig. 4*B* and Table 1), and mass spectrometry confirmed product formation (*m*/*z* = 267) and evidence for a 2,3-epoxide (*m*/*z* = 251). These different reactivities of the 5-OH-Trp and 5-MeO-Trp substrates align with the data for l-Trp and 1-Me-l-Trp as above and are consistent with a radical mechanism being used (presumably for all substrates; Scheme 1).

##### Indole-3-propionic Acid

Oxidation of IPA has never been reported for a heme dioxygenase, but our data support oxidation of this substrate. On reaction of ferrous hIDO with O_2_ and IPA, a ternary [Fe(II)-O_2_, IPA] species forms normally (λ_max_ = 415, 543, and 577 nm; data not shown) after which there is a long lag phase of 50 s during which no product formation occurred (at 321 nm). An intermediate was observed (data not shown), and its decay coincided with product formation (the latter was confirmed by LC-MS, which detected *m*/*z* = 222 as well as *m*/*z* = 206 for the corresponding epoxide). We interpret this to mean that the rate-limiting steps are different from the other substrates examined above so that Compound II does not accumulate, but product formation is still possible. The ammonium ion is presumed (23) to facilitate formation of a ferric superoxide complex (Scheme 1) through hydrogen bonding to the bound oxygen in the ternary complex. We interpret the altered kinetics for IPA (very long lag phase) as being consistent with a role for the ammonium group in stabilizing the ferric superoxide complex (via the radical pathway).

## Discussion

The mechanism of tryptophan oxidation by the heme-containing dioxygenases is a subject of topical recent debate. A base-catalyzed proton abstraction mechanism (5) was widely reproduced in the literature despite the fact that there was barely any experimental evidence for it. Aside from the fact that the aromatic chemistry of indoles dictates that they cannot react in this way (24), the base-catalyzed mechanism is additionally problematic because there is no change in oxidation state of the heme iron during catalysis, which is out of line with all other O_2_-dependent heme enzymes (*e.g.* P450s and NO synthases) that are known to use ferryl intermediates (Compound I).

There is now general agreement that base-catalyzed proton abstraction does not occur (3, 8–11, 23, 25, 26), although the experimental evidence against it is still rather limited. Our data for S-Trp also provide convincing evidence against base-catalyzed abstraction because we observed Compound II formation (Fig. 4*A*), and this is not possible if (as in the case of S-Trp) there is no proton on the indole NH that can be abstracted.

However, the later stages of the mechanism have not been established. Formation of a Compound II has been observed by Raman spectroscopy (8, 9) but only for IDO and not tryptophan 2,3-dioxygenase and only for one substrate (l-Trp). Kinetic identification of the ferryl intermediates in IDO (or indeed in tryptophan 2,3-dioxygenase) has proved to be very difficult. This is in part because the ferrous-oxy species are often unstable so that spectra of pure ferrous-oxy species have not been easy to obtain and in part because differentiating the spectrum of an incompletely formed (impure) ferrous-oxy species from that of a ternary complex or an intermediate Compound II species is not straightforward.

**FIGURE 5.**
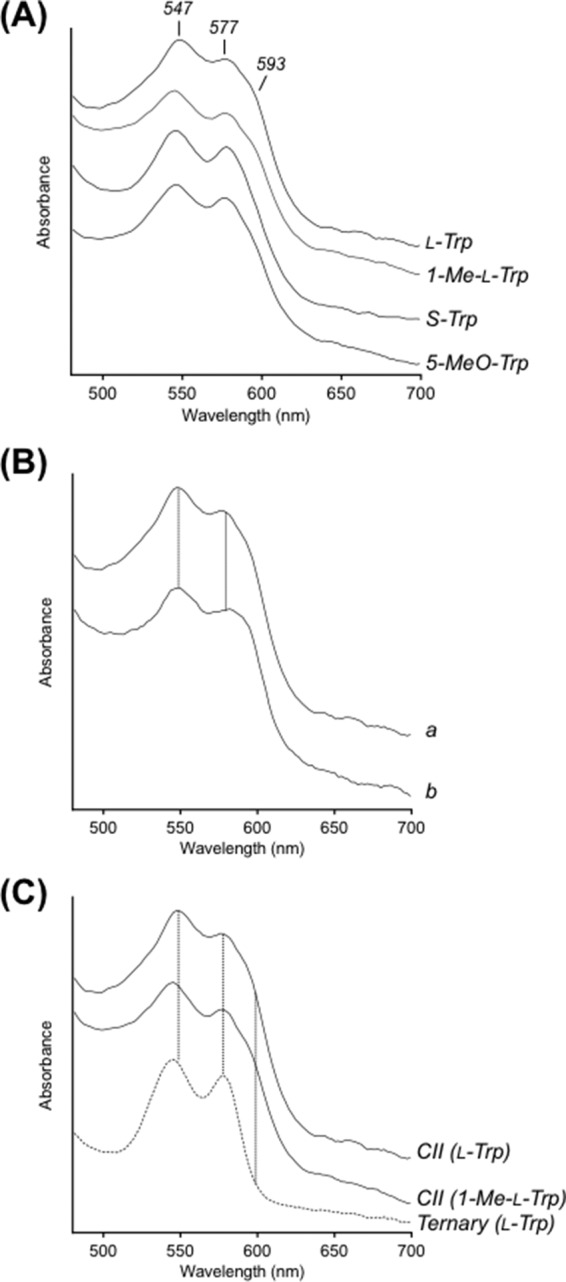
**Analysis and comparison of Compound II spectra.** *A*, overlay of the Compound II spectra obtained during turnover with l-Trp, 1-Me-l-Trp, S-Trp, and 5-MeO-Trp; similar spectra were observed for 5-F-Trp, 5-Me-Trp, and d-Trp (Table 1). *B*, overlay of the spectrum of Compound II (spectrum *a*) observed during turnover of l-Trp with that of the Compound II species (spectrum *b*) formed directly on reaction of ferrous IDO with H_2_O_2_. *C*, overlay of the spectra of Compounds II (*CII*) (*solid lines*) observed during turnover of l-Trp and 1-Me-l-Trp with that of the ternary [Fe-O_2_-Trp] complex (*dashed line*) isolated in the same turnover experiment with l-Trp, showing the differences in the spectroscopic features and the wavelength maxima. Note the absence of the 593 nm shoulder in the ternary complex.

Our kinetic experiments allow us to differentiate all three species. We demonstrate clean formation of ferrous-oxy IDO (λ_max_ = 416, 543, and 577 nm) under non-turnover conditions (Fig. 2*A*); these spectra are in exact agreement with previously reported spectra for ferrous-oxy IDO prepared using different conditions (27). We show that the spectrum of this ferrous-oxy species is subtly different from that of the ternary [Fe(II)-O_2_, Trp] complex (λ_max_ = 413, 543, and 577 nm) isolated during turnover conditions (Fig. 2*B*); we assign these minor differences as being due to binding of l-Trp to the Fe(II)-O_2_ species. Furthermore, during IDO-catalyzed oxidation of l-Trp, we identified a Compound II intermediate (λ_max_ = 416, 547, 577, and 593^sh^ nm) (Fig. 1*A*) that is spectroscopically distinct from either the ferrous-oxy species or the ternary [Fe-O_2_-Trp] complex. This same Compound II intermediate was also observed during turnover of 1-Me-Trp, S-Trp, and 5-MeO-Trp by ferrous hIDO (Figs. 1, *A* and *B*, and 4, *A* and *B*). Fig. 5*A* demonstrates the consistency of these Compound II spectra identified in each of these turnover experiments with the four different substrates with the feature at 593 nm present in all cases. The spectra of these Compound II species observed under turnover conditions are similar to those observed for a “genuine” ferryl species formed in IDO directly but under different conditions (by reaction of ferrous enzyme with peroxide) (Fig. 5*B*) and are subtly different from spectra of the ternary complexes observed in the same turnover experiments (as shown in Fig. 5*C*).

In summary, we detected a transient intermediate, assigned as a Compound II, in the reactivity of IDO with a number of different substrates. The evidence for the range of compounds examined herein strongly suggests that Compound II is the intermediate for all IDO-catalyzed reactions and that oxidation of all substrates by IDO occurs by a common mechanism. IDO has a very wide substrate specificity, much wider than that of tryptophan 2,3-dioxygenase, so this information will provide a basis for development of heme dioxygenases as therapeutic targets as an understanding of mechanism underpins structure-based inhibitor design.

## Author Contributions

E. L. R. and S. H. conceived and coordinated the work. E. S. B. and J. B. collected the kinetic data. E. S. B. and J. B. analyzed the kinetic data with assistance from E. L. R. and S. H. M. L. was responsible for collecting and analyzing the mass spectrometry data. E. L. R. coordinated writing of the paper with assistance from all authors.

## Footnotes

3 Although Compound II does not accumulate during oxidation of 5-OH-l-Trp by hIDO, formation of this intermediate (λ_max_ = 413, 546, and 593^sh^ nm) was observed on reaction of ferrous hIDO (2.5 μm) with 5 eq of H_2_O_2_ and 5-OH-Trp (50 μm).

4 It is probably the case that these relative rates of activity of 5-OH-l-Trp and 5-MeO-Trp apply for other heme dioxygenases, although there are few reports. For example, in the case of IDO2 (22), there is no reported oxidation of 5-OH-Trp, but oxidation of 5-MeO-Trp (*k*_cat_ = 0.161 s^−1^) is similar to l-Trp (*k*_cat_ = 0.103 s^−1^).

## Footnotes

2 The abbreviations used are:

IDO: indoleamine 2,3-dioxygenase
sh (superscript): shoulder
NFK: *N*-formylkynurenine
hIDO: human IDO
IPA: indole-3-propionic acid
S-Trp: β-[3-benzo(*b*)thienyl]-l-alanine.
